# Social integration and risk of mortality among African-Americans: the Jackson heart study

**DOI:** 10.1007/s00127-023-02485-1

**Published:** 2023-05-16

**Authors:** Harold H. Lee, Sakurako S. Okuzono, Claudia Trudel-Fitzgerald, Peter James, Hayami K. Koga, Mario Sims, Francine Grodstein, Laura D. Kubzansky

**Affiliations:** 1 Department of Social and Behavioral Sciences, Harvard T.H. Chan School of Public Health, Boston, MA USA; 2 Lee Kum Sheung Center for Health and Happiness, Harvard T.H. Chan School of Public Health, Boston, MA USA; 3 Department of Epidemiology, University of North Carolina at Chapel Hill, Chapel Hill, NC USA; 4 Department of Population Medicine, Harvard Medical School and Harvard Pilgrim Health Care Institute, Boston, MA USA; 5 Department of Environmental Health, Harvard T.H. Chan School of Public Health, Boston, MA USA; 6 Department of Social Medicine, Population and Public Health, School of Medicine, University of California at Riverside, Riverside, CA USA; 7 Rush Alzheimer’s Disease Center, Rush Medical College, Chicago, IL USA; 8 Present Address: Department of Biobehavioral Health, College of Health and Human Development, The Pennsylvania State University, 124 Biobehavioral Health Building, University Park, PA 16802 USA; 9 Present Address: Department of Psychology at Université du Québec à Trois-Rivières, Trois-Rivières, QC Canada; 10 Present Address: Research Center of Institut Universitaire en Santé Mentale de Montréal, Montreal, QC Canada

**Keywords:** Social integration, Social networks, Social isolation, Social exclusion, Longevity, Mortality, African-American

## Abstract

**Objective:**

Evidence suggests that greater social integration is related to lower mortality rates. However, studies among African-Americans are limited. We examined whether higher social integration was associated with lower mortality in 5306 African-Americans from the Jackson Heart Study, who completed the Berkman-Syme Social Network Index in 2000–2004 and were followed until 2018.

**Methods:**

We estimated hazard ratios (HR) of mortality by categories of the Social Network Index (i.e., high social isolation, moderate social isolation [reference group], moderate social integration, high social integration) using Cox proportional hazard models. Covariates included baseline sociodemographics, depressive symptoms, health conditions, and health behaviors.

**Results:**

Compared with moderate isolation, moderate integration was associated with an 11% lower mortality rate (HR = 0.89, 95% confidence interval [CI] 0.77, 1.03), and high integration was associated with a 25% lower mortality rate (HR = 0.75, 95% CI 0.64, 0.87), controlling for sociodemographics and depressive symptoms; compared with moderate isolation, high isolation was related to a 34% higher mortality rate (HR = 1.34, 95% CI 1.00, 1.79). Further adjustment of potential mediators (health conditions and health behaviors) only slightly attenuated HRs (e.g., HR_moderate integration_ = 0.90, 95% CI 0.78, 1.05; HR_high integration_ = 0.77, 95% CI 0.66, 0.89).

**Conclusion:**

Social integration may be a psychosocial health asset with future work needed to identify biobehavioral processes underlying observed associations with mortality among African-Americans.

**Supplementary Information:**

The online version contains supplementary material available at 10.1007/s00127-023-02485-1.

## Introduction

Emerging empirical studies from wild mammals consistently show a positive relationship between social connection and survival [1–3], suggesting the link between social connection and health is an evolutionarily conserved mechanism. Consistent with this observation, a large body of epidemiological research in humans suggests that both quantity and quality of social connections are related to mortality risk. Notably, there are various types of social connections. In a meta-analysis of 148 studies that examined mortality rates associated with various indicators of social connectedness in humans (e.g., quality or number of social relationships, perceived loneliness), mortality was most strongly associated with measures of social integration [4].

Social integration refers to structural (vs. functional or perceived) aspects of social connection, and has been conceptualized based on an individual’s social network’s characteristics, size, and contact frequency, such as marital status, contacts with close friends and relatives, and participation in religious and group activities [5]. These various ways of being socially integrated, in turn, influence health through many multifaceted downstream factors, many of which have systemic effects on health, such as social support, access to knowledge, material, or other resources, and biobehavioral factors, to name a few (for the conceptual model for social integration and health, see Fig. 1 in Berkman et al. [5]). While pathways via one or two of these factors could be blocked in certain populations, for example, due to aspects of their sociocultural milieu (e.g., limited access to resources due to having lower socioeconomic position; discrimination and racism in African Americans [6, 7]), it is unlikely that most or all of these pathways could be uniformly compromised in such a way as to block all potential health benefits. Thus, the theoretical model suggests that social integration’s impact on health is likely to be evident in most populations.

**Fig. 1:**
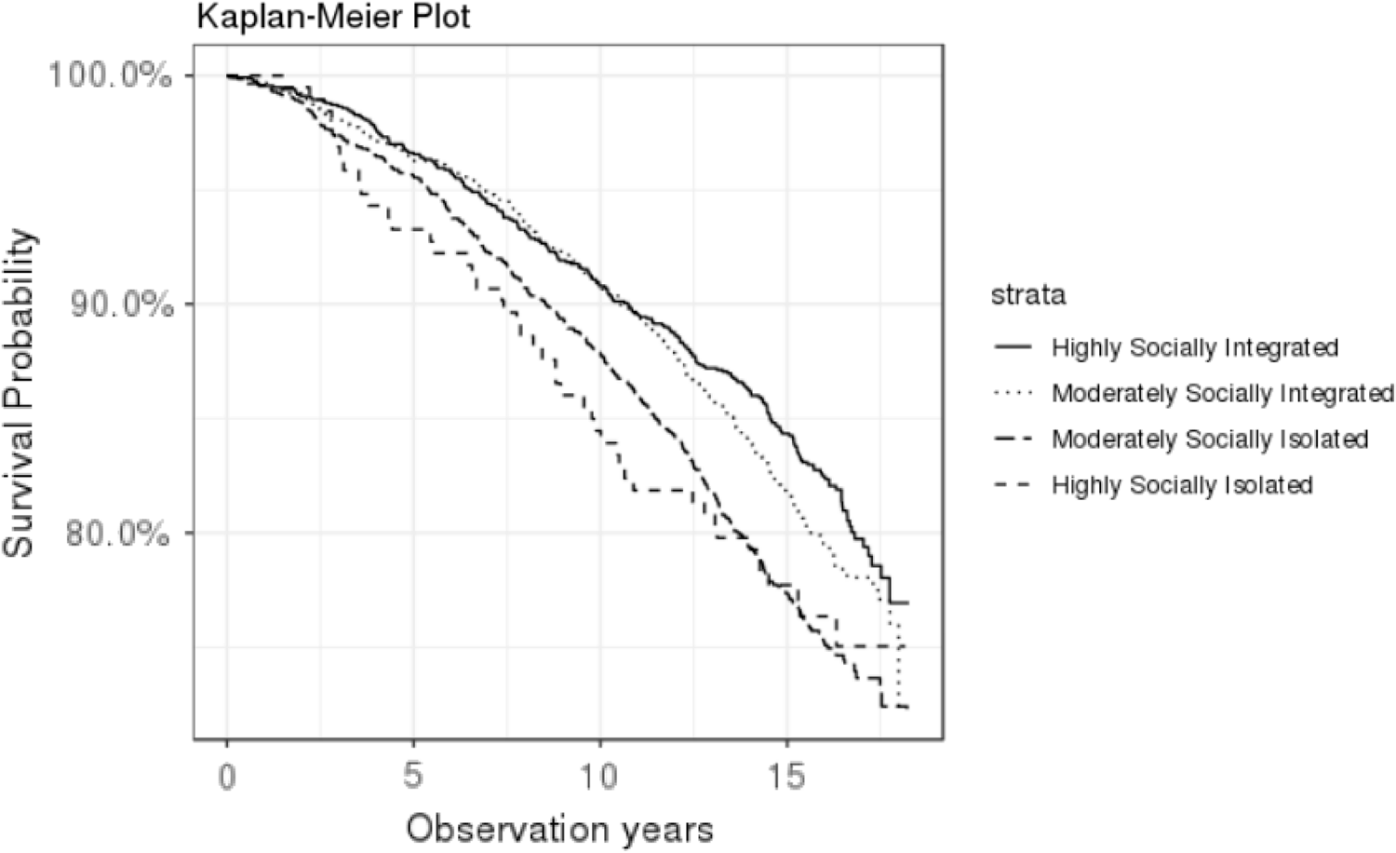
Kaplan–Meier survival curves for participants in the Jackson Heart Study 2001–2018, stratified by level of social integration

However, empirical studies in African Americans have shown mixed results. In this nascent body of research, some work suggests the relationship may differ in African-Americans compared to Whites [8–10]. For example, in one study using data from the US National Health Interview Survey, which included African-Americans and Whites, authors classify participants as “highly isolated”, “moderately isolated”, “moderately integrated”, and “highly integrated”. Higher integration was associated with lower mortality in a dose–response manner among Whites; among African-Americans, the protective association with mortality was observed only at the highest social integration level [9]. Notably, this study had a substantially smaller sample size for African-Americans (*n* = 4201) than Whites (*n* = 20,217), limiting the ability to detect relations across the full distribution of social integration in African-Americans. Another study followed 20,668 African-Americans in the Cancer Prevention Study II for 40 years and found higher social integration was associated with a lower mortality rate in African-Americans [11]. However, despite the large sample size of Cancer Prevention Study II, participants in this study were generally well-educated (e.g., 38% are college graduates) and older (69% greater than 60 years), thus further research is needed in broader and more diverse segments of the population. Since age and socioeconomic status may modify the relationship between social integration and health [12], investigating these associations in more socioeconomically diverse African-Americans with a wide age range will add crucial empirical evidence to the currently limited body of research in this population.

We examined the relationship between social integration and mortality using data from the Jackson Heart Study (JHS), a community-based longitudinal cohort comprised of socioeconomically diverse African-American men and women. We speculate that the social integration is a resource that protects health, likely transcending specific sociocultural milieus. Thus, we hypothesized that higher social integration would be associated with lower mortality rates over the follow-up period in this sample of African American adults. Given prior systematic reviews linking depression, health conditions (e.g., hypertension, diabetes) and behaviors (e.g., physical activity, smoking) with both lower social integration and greater risk of mortality [13, 14], these factors were examined as potential confounders. Prior research found differences in the social integration-mortality relationship by sex [11], and JHS is comprised of a socioeconomically diverse population with a wide age range. Thus, we examined sex, age, education, and income as potential effect modifiers.

## Methods

### Study population

At baseline, 5,306 African-American adults ages 20–95 were recruited into JHS, from Hinds, Madison, and Rankin counties in the Jackson, Mississippi, metropolitan area. Participants came from four recruitment pools as follows: community random sample (17%), volunteer sample (22%), the Atherosclerosis Risk in Communities Study sample (30%), and family sample which included individuals who were members of families that already had at least two members enrolled in the study (31%). Recruitment was limited to noninstitutionalized African Americans aged 35 to 84 years, except in the family sample, in which those aged 21 to 34 years were also eligible [15]. Additional details of the design and recruitment are published elsewhere [16–18]. After completing baseline clinical visits (exam1: 2000 to 2004), participants returned for 2 additional examinations (exam2: 2005–2008, and exam3: 2009–2013), and follow-up questionnaires were administered annually by telephone. All participants (*n* = 5306) were followed up through 2018 for mortality status.

In general, missing data was low, on both primary variables of interest and covariates (< 3%). For example, for most components of the social integration measure including marital status, contact with close friends and relatives, and participation in group activities, there were few missing data at < 1%; missing data was higher for church attendance (*n* = 1033; 19% missing). JHS participants who did not versus did provide social integration data did not differ significantly in age (56 vs. 54 years) and body mass index (BMI; 31.7 vs. 31.8 kg/m^2^), but they did have lower income (e.g., 22% vs. 27% are affluent), lower education (e.g., 51% vs 64% attended vocational/trade, or college), and were slightly more likely to engage in unhealthy behaviors (e.g., 16% vs. 12% are current smokers) (Supplemental Table S1). Most covariates had little missing data (< 3%), with somewhat higher missing data for income (15%), cholesterol (13%), diet (10%), and depression status (36%). As all missing data occurred at random, we imputed these missing data in all 4 domains of social integration as well as covariates to retain sample size for our analyses. Thus, all participants were retained for the analytic sample. The Institutional Review Boards approved the study at the [blinded for review]. All participants provided written informed consent. The Institutional Review Board at [blinded for review] approved the current analysis.

### Measures

#### Social integration

At the baseline period, social integration was assessed based on the Berkman-Syme Social Network Index [5], using information from the following four domains: (1) marital status, (2) contact with close friends and relatives, (3) participation in group activities, and (4) participation in religious activities. Following prior work [19], we assigned a score of 0 to those who were least integrated and a score of 3 to those who were most integrated within each domain, as described in more detail below.

Marital status assesses whether participants are married or in a relationship, divorced, never been married, separated, widowed, or not in a relationship. We created a binary variable assigning a score of 3 to those who were married or in a relationship and 0 to all others. Contact with friends and relatives assesses the number of (a) close friends and (b) close relatives participants can talk to; response options for each include the following: ‘none’, ‘1 to 2,’ ‘3 to 5,’ ‘6 to 9,’ and ‘10 or more.’ For an overall measure, we assigned the median value to each response (‘none’ = 0, ‘1 to 2’ = 1.5, ‘3 to 5’ = 4, ‘6 to 9’ = 7.5, ‘10 or more’ = 10), then summed across friends and relatives to obtain a score ranging from 0–20; we then categorized the score as follows: 0 = none; 1 = 1–2; 2 = 3–5; 3 = 6 or more. Participation in group activities was assessed by summing the number of groups to which participants reported belonging, and then categorizing this continuous score: 0 = 0; 1 = 1 group; 2 = 2–3 groups; 3 = 4 or more groups. Participation in religious activities was assessed according to self-reported frequency of church attendance. Following prior work [19], we categorized this as: 0 = not at all; 1 = a few times a year or < once a year; 2 = a few times a month; 3 = nearly every day or at least once a week. We then created a continuous social integration score for all participants by summing scores across domains, for a total score ranging from 0 to 12. To examine threshold effects, this continuous social integration score was also categorized as: highly isolated (0–4), moderately isolated (5–7), moderately integrated (8–9), highly integrated (10 or higher).

#### Mortality

Mortality was assessed using information from the Mississippi Department of Health and the National Death Index. Specifically, JHS obtained mortality status at annual follow-up and from physician and coroner reports, and this information was adjudicated by trained clinical staff. Death certificates were requested from the Mississippi State Department of Health for JHS participants when needed [20]. For example, as part of data collection for mortality, the JHS uses age-specific death lists of African Americans in the tri-county area and requests death certificates from the State Department of Health. For the present analysis, data on mortality were available through May 31, 2018.

#### Covariates

All covariates were obtained at baseline. Demographic measures were self-reported. Based on income, family size, and inflation, JHS investigators classified annual household income into 4 categories: low (less than poverty level), lower-middle (1–1.6 times the poverty level), upper-middle (1.6–3.5 times the poverty level), and affluent (at least 3.5 times the poverty level). We classified education into three categories: vocational/trade school/college or higher, high school graduate/GED, and less than high school. Depressive symptoms were assessed using the 20-item Center for Epidemiologic Studies Depression Scale (CES-D), and a score ≥ 16 was defined as probable depression [21, 22].

The measurements and categorizations of health conditions and health behavior-related factors are detailed in Supplemental Text 1. Briefly, health conditions included prevalent or history of high cholesterol, type 2 diabetes mellitus, hypertension, cardiovascular disease, and cancer. Health behavior-related factors included smoking, physical activity, diet, alcohol consumption, and BMI.

### Statistical analysis

We visually observed the unadjusted relationship between social integration and mortality rate using Kaplan–Meier curves. Then, we used Cox proportional hazards regression to examine the relationship between social integration and mortality, accounting for relevant covariates, with 5 increasingly covariate adjusted models. Respondents were censored (i.e., follow-up ended) after (1) death, (2) loss to follow-up (i.e., could not be found for further evaluation), or (3) the end of follow-up (May 31, 2018). The first model included age. The second model further added sex, income, and education. The third model additionally controlled for depressive symptoms as a potential confounder (and/or intermediate variable), constituting the core model that accounts for key covariates related to mortality and social integration. The fourth and fifth models included health conditions and behaviors that could be confounders or intermediate variables. The fourth model added prevalent/history of high cholesterol, diabetes, hypertension, cardiovascular disease, and cancer. The fifth model further added health behaviors (i.e., smoking, physical activity, alcohol consumption, diet quality) and BMI, constituting the fully adjusted model. We examined whether the proportional hazards assumption of Cox models was violated by Schoenfeld residuals [23], and the proportionality assumption was upheld in the full model (*P* = 0.27). In all models, we included the categorical measure of social integration, with the category ‘moderately isolated’ serving as the reference group to facilitate examining potential effects of either extreme (i.e., highly isolated or highly integrated). To evaluate potential linear trends, *P* values for trend were determined using the continuous social integration score (range 0–12).

With secondary analyses, we tested potential effect modification by sex (male/female), age (binary; age ≥ 55 vs. < 55), education (binary; years of education ≤ 13 vs. > 13), and income (binary; low and low middle vs. middle and affluent), including interaction terms separately in the core model. We also examined each component of the social integration score separately in relation to mortality risk using the same set of models described above. We further conducted several sensitivity analyses. First, we calculated *E*-values [24, 25] to quantify the extent to which an unmeasured confounder would need to be associated with both social integration and mortality to explain away the observed association. Second, to reduce concerns about reverse causation (i.e., underlying illness could lead to changes in social integration) we conducted 2 analyses whereby we: (1) excluded 190 participants who died within 4 years of study baseline (resulting in *n* = 5116); or (2) excluded 837 individuals with cardiovascular disease and cancer at baseline (resulting in *n* = 4470). Third, instead of imputing social integration data, we conducted analyses after excluding 1082 participants missing any components in the social integration measure (resulting in *n* = 4224). Finally, to facilitate more direct comparison with the existing body of research on social integration, we conducted analyses combining “highly isolated” and “moderately isolated”. All analyses were conducted in R Statistical Software (version 1.1456) (16). We conducted multiple imputations (*n* = 10) by chained equations using the MICE package in R and combined results from each imputed dataset using the MITOOL package in R [26].

## Results

The mean social integration score was 8.19. Table 1 shows the distributions of covariates at baseline by levels of social integration. Participants had a mean age of 55 years (range 20–95 years), and 64% were women. At baseline, 58% of participants were married or living together, 53% reported having 6 or more close friends/relatives, 79% reported attending religious activities once a week or more, and 11% reported belonging to 4 or more social/church groups. The continuous social integration score was negatively skewed, indicating most of the JHS participants reported relatively high social integration (Supplemental Figure S1). Compared to those who were moderately isolated, those categorized as highly integrated reported higher income and education, were less likely to be depressed and were less likely to be a current smoker.

**Table 1:** Baseline Characteristics of Participants by Categories of Social Integration Score^a^, Jackson Heart Study, 2000–2004 (*N* = 5306)

	Highly isolated (0–4)	Moderately isolated (5–7)	Moderately integrated (8–9)	Highly integrated (10–12)
	(*N* = 193)	(*N* = 1410)	(*N* = 1286)	(*N* = 1332)
Demographic factors				
Age, years, M (SD)	48.3 (13.2)	54.7 (13.5)	54.3 (12.1)	55.6 (11.6)
Female, %	112 (58.0%)	1041 (73.8%)	801 (62.3%)	773 (57.9%)
Income,^b^ %				
Poor	64 (33.2%)	268 (19.0%)	115 (8.9%)	68 (5.1%)
Lower-middle	41 (21.2%)	383 (27.2%)	253 (19.7%)	186 (13.9%)
Upper-middle	29 (15.0%)	349 (24.8%)	347 (27.0%)	378 (28.3%)
Affluent	26 (13.5%)	230 (16.3%)	391 (30.4%)	473 (35.4%)
Educational level, %				
Less than high school	40 (20.7%)	287 (20.4%)	205 (15.9%)	156 (11.7%)
High school graduate/GED	47 (24.4%)	330 (23.4%)	248 (19.3%)	205 (15.4%)
Vocational/trade, or college	105 (54.4%)	790 (56.0%)	832 (64.7%)	973 (72.9%)
Mental health				
Depressive symptoms, %	55 (28.5%)	257 (18.2%)	184 (14.3%)	150 (11.2%)
Baseline health conditions				
High cholesterol,^c^ %				
Poor	25 (13.0%)	179 (12.7%)	194 (15.1%)	160 (12.0%)
Intermediate	57 (29.5%)	517 (36.7%)	437 (34.0%)	500 (37.5%)
Ideal	89 (46.1%)	546 (38.7%)	515 (40.0%)	530 (39.7%)
Type 2 mellitus, %	48 (24.9%)	342 (24.3%)	270 (21.0%)	309 (23.1%)
Hypertension, %	85 (44.0%)	825 (58.5%)	733 (57.0%)	731 (54.8%)
CVD history, %	21 (10.9%)	158 (11.2%)	128 (10.0%)	118 (8.8%)
Cancer history, %	10 (5.2%)	52 (3.7%)	59 (4.6%)	67 (5.0%)
Health behaviors				
Smoking status, %				
Current smoker	60 (31.1%)	213 (15.1%)	166 (12.9%)	84 (6.3%)
Former smoker	31 (16.1%)	256 (18.2%)	246 (19.1%)	262 (19.6%)
Never smoked	101 (52.3%)	932 (66.1%)	869 (67.6%)	980 (73.4%)
Physical activity,^d^ %				
Poor	109 (56.5%)	751 (53.3%)	612 (47.6%)	560 (41.9%)
Intermediate	49 (25.4%)	449 (31.8%)	394 (30.6%)	450 (33.7%)
Ideal	35 (18.1%)	210 (14.9%)	280 (21.8%)	325 (24.3%)
Diet,^e^ %				
Poor	110 (57.0%)	700 (49.6%)	670 (52.1%)	652 (48.8%)
Intermediate	50 (25.9%)	539 (38.2%)	485 (37.7%)	565 (42.3%)
Ideal	0 (0%)	20 (1.4%)	9 (0.7%)	16 (1.2%)
Alcohol,^f^ %				
Excessive drinker	21 (10.9%)	67 (4.8%)	34 (2.6%)	19 (1.4%)
Moderate drinker	107 (55.4%)	520 (36.9%)	504 (39.2%)	482 (36.1%)
No drinker	55 (28.5%)	789 (56.0%)	711 (55.3%)	798 (59.8%)
Body mass index, M (SD)	31.3 (8.28)	32.2 (7.77)	31.7 (7.15)	31.3 (6.57)

Percentage may not add up to 100% due to missing values

This table was created using data prior to imputing social integration variable

^a^Social integration was derived from Berkman–Syme Social Network Index, a continuous score ranging from 0 to 12

^b^Income status derived from family income and family size and adjusted for inflation

^c^Poor: total cholesterol $\ge$ 240 mg/dL; Intermediate: 200–239 mg/dL, or if treated (< 200 mg/dL); Ideal: < 200 mg/dL

^d^Poor: 0 min of moderate or vigorous physical activity; Intermediate: 1–149 min/wk of moderate physical activity, 1–74 min/wk of vigorous physical activity, or 1–49 min/wk of combined moderate and vigorous physical activity; and Ideal: ≥ 150 min/wk of moderate physical activity; or ≥ 75 min/wk of vigorous physical activity; or ≥ 150 min/wk of combined moderate and vigorous physical activity

^e^Diet quality was assessed by evaluating ideal consumption levels for 5 dietary components (fruits and vegetables:$\ge$ 4.5 cups/day; fish: > 3.5 oz, twice per week; sodium: < 1500 mg/day; sugary beverages: < 450 kcal/wk and whole grains: $\ge$ 3 servings/day). The food frequency questionnaire was then categorized into three groups based on the number of components that meet the guideline. Poor: 0–1 components; Intermediate: 2–3 components; Ideal: 4–5 components

^f^Poor: > 7 drinks/wk for women and > 14 for men; Intermediate: zero drinks/wk; and Ideal: 1–7 drinks/wk for women and 1–14 for men

### Social integration and mortality

Over approximately 18 years of follow-up, 23% of participants died (*n* = 1238). Overall, we observed strong dose–response associations between higher social integration levels and lower mortality rates in unadjusted Kaplan–Meier curves (Fig. 1). These trends remained largely unchanged in findings from multivariable proportional hazards analyses (Table 2). Compared to moderately isolated individuals, those who were more integrated had lower hazard ratios (HRs) while those who were more isolated had higher HRs of mortality over the follow-up. For example, in the core model (Model 3), the highly integrated group was related to a 25% reduced hazard for death during follow-up. Adding health conditions and factors related to health behaviors to the core model did not yield a substantial attenuation with the association indicating 23% reduced hazard. Reduced mortality rates were also observed for the moderately integrated group, though the association did not reach statistical significance (e.g., core model: HR = 0.90, 95% CI 0.78–1.05). Conversely, compared to individuals who were moderately isolated, those who were highly isolated had an HR of 1.35 (95% CI 0.98–1.86) for death during the follow-up in the core model. However, after adding health conditions and health-behavior-related factors to the core model, the primary association was substantially attenuated (HR = 1.19, 95% CI 0.88–1.62); of note, relatively few participants were highly isolated (*n* = 242). The relationship between social integration and mortality was not modified by sex, age, education, or income (Supplemental Table S2).

**Table 2:** Hazard ratios for associations of social integration with all-cause mortality, Jackson Heart Study, 2000–2018 (*N* = 5306)

Exposure	Person-years	Cases	Model 1^a^		Model 2^b^		Model 3^c^		Model 4^d^		Model 5^e^	
			HR	95%CI	HR	95%CI	HR	95%CI	HR	95%CI	HR	95%CI
Berkman–Syme social network index												
Highly integrated	20,031	246	0.70	0.61, 0.81	0.75	0.65, 0.88	0.75	0.65,0.88	0.74	0.64, 0.87	0.77	0.66, 0.90
Moderately integrated	19,195	268	0.88	0.76, 1.01	0.90	0.78, 1.05	0.90	0.78,1.04	0.90	0.78,1.04	0.91	0.79, 1.06
Moderately isolated	20,468	351	1.00	Ref	1.00	Ref	1.00	Ref	1.00	Ref	1.00	Ref
Highly isolated	2,752	46	1.73	1.28, 2.35	1.35	0.98, 1.86	1.33	0.97,1.82	1.29	0.95, 1.74	1.19	0.88, 1.62
P for trend			< 0.001		< 0.001		< 0.001		< 0.001		< 0.001	

*CI* confidence interval, *Ref*. reference, *HR* hazard ratio, *CES-D* center for epidemiologic studies depression scale

Model 1^a^ Age adjusted

Model 2^b^ Model 1 + sociodemographic conditions (sex, income, and educational status)

Model 3^c^ Model 2 + depression symptom measured by CES-D

Model 4^d^ Model 3 + health conditions (cholesterol, diabetes, hypertension, cardiovascular disease, and cancer)

Model 5^e^ Model 4 + health behaviors (smoking, physical activity, diet, alcohol consumption, and body mass index)

### Subcomponents of social integration and mortality

In Cox proportional hazard models assessing the subcomponents of social integration separately, greater social integration in all 4 domains was associated with lower mortality in the models controlling only for age (Table 3). Except church attendance, all associations remained significant after further controlling for sociodemographic factors and depressive symptoms. Specifically, in the core models, being married/partnered (vs. not married/not in partnership) was associated with an HR of 0.80 (95% CI 0.71–0.91); having contacts with 6 or more close friends/relatives (vs. 0–2 contacts per month) was associated with an HR of 0.69 (95% CI 0.57–0.85); the highest vs. lowest level of group attendance was associated with an HR of 0.78 (95% CI 0.63–0.96) for mortality over follow-up.

**Table 3:** Hazard ratios for associations of social integration subcomponents with all-cause mortality, Jackson Heart Study, 2000–2018 (*N* = 5306)

Exposure^a^	Person-years	Cases	Model 1^b^			Model 2^c^		
			HR	95%CI	P for Trend	HR	95%CI	P for Trend
Component of the social network index^a^								
Marital status								
Married/in partnership	45,629	618	0.81	0.73, 0.91	NA	0.80	0.71, 0.91	NA
Not married/not in partnership	31,801	634	1.00	Ref		1.00	Ref	
Contact with close friends/relatives								
6 or more contacts/month	40,583	699	0.60	0.49, 0.73	0.015	0.69	0.57, 0.85	0.038
3–5 contacts/month	30,805	439	0.61	0.50, 0.75		0.71	0.57, 0.88	
0–2 contacts/month	5854	110	1.00	Ref		1.00	Ref	
Group attendance								
4 or more group attendance	48,652	107	0.63	0.51, 0.77	< 0.001	0.78	0.63, 0.96	0.010
2–3 group attendance	19,154	273	0.77	0.67, 0.88		0.88	0.76, 1.01	
0–1 group attendance	9270	869	1.00	Ref		1.00	Ref	
Church attendance								
More than once a week	50,278	766	0.75	0.57, 1.00	0.021	0.97	0.73, 1.29	0.748
A few times a month	8571	104	0.86	0.62, 1.19		1.00	0.72, 1.39	
Less than few times a year	4375	51	1.00	Ref		1.00	Ref	

*CI* Confidence Interval, *Ref*. Reference, *HR* Hazard Ratio, *CES-D* Center for Epidemiologic Studies Depression Scale

^a^While each component of social network index are presented in the same table, they represent distinct analyses

^b^Adjusted for age

^c^Adjusted for age, sex, educational status, income, and depression symptom measured by CES-D

### Sensitivity analyses

Sensitivity analyses suggest associations between social integration and mortality are unlikely to be explained by unmeasured confounding. The *E*-value in the core model comparing moderately vs. highly integrated groups was 1.74 [24], suggesting an unmeasured confounder would need to have a minimum HR of 1.74 with both higher social integration and lower mortality (above and beyond the measured covariates) to fully explain away the observed association [25]. After excluding those who died within 4 years from baseline, estimates remained largely unchanged from the primary analyses (Supplemental Table S3). Findings were largely consistent in analyses excluding individuals with cardiovascular disease and cancer at baseline (Supplemental Table S4), in analyses in which we did not impute missing observations for church attendance (Supplemental Table S5), and in analyses in which we combined “highly isolated” and “moderately isolated” groups and used them as the reference group (Supplemental Table S6).

## Discussion

In a prospective, community-based cohort of socioeconomically diverse African-American men and women with a broad age range, we found higher social integration was robustly related to reduced risk of premature mortality in a dose–response manner over ~ 18 years of follow-up. Compared with those who were moderately isolated, being moderately integrated was associated with 11% lower mortality rates, and being highly integrated with 25% lower mortality rates over the follow-up, after controlling for core confounders. Compared with those who were moderately isolated, being highly isolated was related to a 34% higher mortality rate, controlling for core confounders. Further adjustment for health conditions and health behaviors, which may be confounders or intermediate variables on the pathway from social integration to mortality, only slightly attenuated most associations. In analyses assessing social integration domains separately, higher social integration in each domain except church attendance was associated with lower risk of mortality even after adjustment for core confounders. Taken together, these findings suggest that social integration may be a psychosocial asset that deserves further consideration as a possible intervention target to reduce mortality among African-Americans.

Our findings generally converge with the body of evidence in predominantly White populations suggesting that higher social integration is related to reduced mortality risk. Although some studies hinted that the social integration-mortality link differed between Whites and African-Americans [8–10], in all of these studies the sample size was substantially smaller for African-American than for Whites. Our findings align with several other studies that reported similar associations of social integration and mortality across Whites and African-Americans that included larger numbers of African-Americans [11, 27]. For example, in the Cancer Prevention Study II (CPS-II), in which 20,668 well-educated and relatively older (50–79 years) African-Americans were followed for up to 40 years, the most isolated Black men had a 60% higher mortality rate than the least isolated Black men, while Black women had an 84% higher rate [11]. Of note, compared to the present study, risk estimates regarding the association of the social isolation with mortality were larger in the meta-analysis with individuals of predominantly European descent and in the study of African Americans in CPS-II (i.e., hazard ratios for high social isolation: 2.00 in CPS-II [11] and 1.92 in the meta-analysis [4] > vs. 1.31 in the present study). While the differences in effect sizes between these studies and the present one partially due to heterogeneity in which social integration measures were used and different scaling of these measures, the subcomponents examined in each study are conceptually similar and the general patterns of results, including a dose–response relationship, is consistent across studies. Moreover, although African-Americans in JHS represent a broader spectrum of education levels and ages compared to CPS-II participants, the social integration–mortality associations did not differ by education or age, suggesting these associations are not only evident among those who are more socioeconomically advantaged. In subcomponent analyses, we found associations with reduced mortality risk for 3 of the 4 domains of social integration, suggesting there may not be only one component driving these associations. These results align with previous studies among African-Americans, demonstrating favorable associations for contact with friends, marital status, and the number of groups with other health-related outcomes (e.g., health behavior, health care practices, coping/mental health) [28–32]. Given prior studies showed that higher church attendance is associated with lower mortality rates [33, 34], the null association in the present study is unexpected and may be due to a lack of variation in church attendance (i.e., 79.1% reported attending religious activities once a week or more).

Drawing on a conceptual framework developed after systematically reviewing relevant studies, Hodgson et al. recently proposed 4 major pathways that could underlie the association of social integration and mortality [14]. These include physiological (e.g., hypothalamic–pituitary–adrenal axis and inflammation), psychological (e.g., anxiety and cognitive decline), sociological (e.g., access to health care, health literacy), and behavioral pathways (e.g., physical activity and smoking). For example, empirical studies show that increases in social integration levels were associated with reduced inflammation and more frequent healthy dietary choices. While social integration was associated with several factors in the current study (e.g., diet and physical activity), the social integration-mortality associations were only slightly attenuated when including them in the models. Future research should further evaluate the role of these and other mechanisms we could not assess in the current study (e.g., inflammation).

The associations between social integration and mortality were not modified by sex, age, education, or income. Combining this observation with the larger body of research on social integration and health in Whites and African-Americans, such findings may suggest that once individuals become socially integrated, relations with health may be positive across racial/ethnic and sociodemographic groups regardless of how one achieves social integration. Aligning with this observation, emerging results from wild mammals consistently show a positive relationship between social integration and survival with remarkably similar effect sizes as reported in humans (i.e., odds ratios ranging from 1.23 to 1.72) [1–3], suggesting the link between social integration and health is an evolutionarily conserved mechanism. However, since structural factors can pattern social network structures and functions [35, 36], different racial/ethnic groups may have different paths to or experiences of social networks. Thus, network characteristics might differ for African-Americans relative to individuals from other sociocultural backgrounds like Hispanic Americans and non-Hispanic White Americans, as well as non-Westerners. For example, compared to White Americans, some work suggests African Americans may have more kin than friends in their networks [37–39] and rely more on kin-centered networks for support [40–43]. In non-kin social networks, religious involvement and ties to one’s church community are particularly salient in African American culture [35, 44]. Such sociocultural contexts could be more explicitly considered when designing studies to assess how social integration in different groups may influence health. Extrapolating from this, the degree of “kin-centeredness” is potentially a useful factor to consider when evaluating effects of social integration, given humans are genetically predisposed to be more altruistic to those who share a higher proportion of genomic information with themselves (i.e., genetic relatives) [45, 46] while culture might also play a central role on individuals’ tendency to engage in prosocial behaviors with non-kin or strangers [47].

Our study has several limitations. First, confounding is always a limitation in observational studies. However, we did control for a wide range of potential confounders, including depressive symptoms, and the calculated *E*-values suggested that observed associations were relatively robust to unmeasured confounding. Reverse causation is possible, however, in sensitivity analyses where we excluded those who died early in the follow-up or with chronic diseases at baseline, findings remained highly consistent. The JHS was conducted in a single metropolitan area in the southern United States, where African Americans are more concentrated compared to other cities. As a result, levels of social integration may differ from those found in other regions. In addition, according to the US census, there are more female than male African Americans in the general population; JHS also includes more women than men [48]. Further, the higher proportion of female participants in the JHS is due in part to the strategy of supplementing the random sample with community volunteers who met specific criteria based on age, sex, and socioeconomic status [49]. This approach was necessary to overcome the challenges of cost and low response rates associated with random sampling. Finally, we were unable to investigate all facets of social integration given the available data. For example, some individuals with less social integration may prefer and find comfort in solitude. Future studies may investigate such effect heterogeneity by individual characteristics in greater detail. However, our study also has several important strengths. We examined the social integration-mortality relationships in a large and socioeconomically heterogeneous sample of African-American men and women. Moreover, we had a substantial follow-up period and accounted for numerous potential confounders and intermediate factors, including some that were clinically assessed.

In summary, higher levels of social integration were associated with reduced mortality risk among African-American adults in a dose–response manner over ~ 18 years of follow-up. Our findings extend prior research by testing if associations hold in a diverse sample of African-Americans regarding socioeconomic status and age. Relatedly, in the present study, the association between social integration and mortality did not differ by sex, age, education, or income, suggesting social integration may be an essential psychosocial asset that promotes health regardless of either race/ethnicity and sociodemographic factors. Thus, social integration may be an intervention target to evaluate further. However, since the specific ways through which individuals acquire and maintain high social integration may differ based on sociocultural background, it is important to recognize approaches to fostering social integration may need to differ as well depending on which groups are targeted for intervention. Recognizing key strengths and assets within diverse groups may provide additional critical levers for improving population health.

## Supplementary Information

Below is the link to the electronic supplementary material.

Supplementary file1 (DOCX 58 KB)

## Data Availability

The data used in this study were obtained from the Jackson Heart Study (JHS), which is supported and conducted in collaboration with Jackson State University (HHSN268201800013I), Tougaloo College (HHSN268201800014I), the Mississippi State Department of Health (HHSN268201800015I) and the University of Mississippi Medical Center (HHSN268201800010I, HHSN268201800011I and HHSN268201800012I) contracts from the National Heart, Lung, and Blood Institute and the National Institute for Minority Health and Health Disparities. The JHS data are available to qualified researchers upon request from the JHS Coordinating Center (https://www.jacksonheartstudy.org). Restrictions may apply to the availability of these data, which were used under license for the current study, and so are not publicly available. However, access to the data can be requested through the JHS Coordinating Center, subject to the JHS Data Use and Publication Policy. Any publications resulting from the use of JHS data should include an acknowledgment of the source of the data and funding support.
