# Supplementary material for: Social integration and risk of mortality among African-Americans: the Jackson heart study
Source: Soc Psychiatry Psychiatr Epidemiol. 2023 May 16;58(9):1317–27. doi: 10.1007/s00127-023-02485-1 (PMC10423160; doi:10.1007/s00127-023-02485-1)
Supplement: Supplementary file 1 — Supplementary file1 (DOCX 58 KB) [file 127_2023_2485_MOESM1_ESM.docx]

**Supplement Material**

**Social integration and risk of mortality among African Americans: The Jackson Heart Study**

Harold H. Lee, Sakurako S. Okuzono, Claudia Trudel-Fitzgerald, Peter James, Hayami K. Koga, Mario Sims, Francine Grodstein, and Laura D. Kubzansky

**Table of Contents**

[**Supplemental Table S1.** Baseline Characteristics of Participants by Initial Missingness of Components of the Social Integration Score, Jackson Heart Study, 2000-2004 (N=5,306) 2](#_Toc95215597)

[**Supplemental Table S2.** Effect Modification by Sex, Age, Education, and Income in the Association Between Social Integration and Mortality, Jackson Heart Study, 2000-2018 4](#_Toc95215598)

[**Supplemental Table S3.** Hazard Ratios for Associations of Social Integration With All-Cause Mortality After Excluding Participants who Died within 4 years from Baseline. Jackson Heart Study, 2000-2018 (n=5,116, n_death_ = 1,065) 5](#_Toc95215599)

[**Supplemental Table S4**. Hazard Ratios for Associations of Social Integration With All-Cause Mortality After Excluding Participants who had Cardiovascular Disease or Cancer at Baseline. Jackson Heart Study, 2000-2018 (n=4,470, n_death_ = 837) 6](#_Toc95215600)

[**Supplemental Table S5**. Hazard Ratios for Associations of Social Integration With All-Cause Mortality Without Imputing Missing in the Social Integration Composite, Jackson Heart Study, 2001-2018 (n=4,224, n_death_ = 911) 7](#_Toc95215601)

[**Supplemental Table S6**. Hazard Ratios for Associations of Social Integration With All-Cause Mortality, when “Highly isolated” and “Moderately isolated” are combined, Jackson Heart Study, 2000-2018 (N=5,306) 8](#_Toc95215602)

[**Supplemental Text 1.** Description of Health Conditions and Health Behavior-related Factors 9](#_Toc95215603)

[**Supplemental Figure S1.** Distribution of the Continuous Social Integration Score, Jackson Heart Study, 2000-2004 11](#_Toc95215604)

To address these comments, could you conduct below analyses and fill in the attached table? (relevant parts are highlighted in yellow).

# **Supplemental Table S1.** Baseline Characteristics of Participants by Initial Missingness of Components of the Social Integration Score, Jackson Heart Study, 2000-2004 (N=5,306)

|  | Provided data for  Social Integration | Missing data for  Social Integration |
| --- | --- | --- |
|  | (N=4221) | (N=1,082) |
| *Demographic Factors* |  |  |
| Age, years, M (SD) | 54.4 (12.6) | 56.0 (13.9) |
| Female, % | 2725 (64.6%) | 640 (59.1%) |
| Income,^a^ % |  |  |
| Poor | 515 (12.2%) | 188 (17.4%) |
| Lower-middle | 863 (20.4%) | 238 (22.0%) |
| Upper-middle | 1102 (26.1%) | 227 (21.0%) |
| Affluent | 1119 (26.5%) | 236 (21.8%) |
| Educational Level, % |  |  |
| Less than high school | 688 (16.3%) | 285 (26.3%) |
| High school graduate/GED | 829 (19.6%) | 235 (21.7%) |
| Vocational/trade, or college | 2698 (63.9%) | 550 (50.8%) |
| *Mental Health* |  |  |
| Depressive symptoms, % | 646 (15.3%) | 123 (11.4%) |
| *Baseline Health Conditions* |  |  |
| High cholesterol,^b^ % |  |  |
| Poor | 556 (13.2%) | 149 (13.8%) |
| Intermediate | 1511 (35.8%) | 344 (31.8%) |
| Ideal | 1680 (39.8%) | 381 (35.2%) |
| Type 2 mellitus, % | 967 (22.9%) | 273 (25.2%) |
| Hypertension, % | 2371 (56.2%) | 623 (57.6%) |
| CVD History, % | 425 (10.1%) | 144 (13.3%) |
| Cancer History, % | 186 (4.4%) | 43 (4.0%) |
| *Health Behaviors* |  |  |
| Smoking status, % |  |  |
| Current smoker | 522 (12.4%) | 170 (15.7%) |
| Former smoker | 795 (18.8%) | 191 (17.7%) |
| Never smoked | 2880 (68.2%) | 699 (64.6%) |
| Physical activity,^c^ % |  |  |
| Poor | 2031 (48.1%) | 584 (54.0%) |
| Intermediate | 1340 (31.7%) | 332 (30.7%) |
| Ideal | 850 (20.1%) | 163 (15.1%) |
| Diet,^d^ % |  |  |
| Poor | 2130 (50.5%) | 548 (50.6%) |
| Intermediate | 1638 (38.8%) | 390 (36.0%) |
| Ideal | 45 (1.1%) | 5 (0.5%) |
| Alcohol,^e^ % |  |  |
| Excessive drinker | 141 (3.3%) | 41 (3.8%) |
| Moderate drinker | 1612 (38.2%) | 409 (37.8%) |
| No drinker | 2351 (55.7%) | 602 (55.6%) |
| Body mass index, M (SD) | 31.8 (7.3) | 31.7 (7.2) |

Percentage may not add up to 100% due to missing values.

^a^ Income status derived from family income and family size and adjusted for inflation.

^b^ Poor: total cholesterol $\geq$240 mg/dL; Intermediate: 200 mg/dL – 239 mg/dL, or if treated (< 200 mg/dL); Ideal: < 200 mg/dL.

^c^ Poor: 0 minutes of moderate or vigorous physical activity; Intermediate: 1–149 min/wk of moderate physical activity, 1–74 min/wk of vigorous physical activity, or 1–49 min/ wk of combined moderate and vigorous physical activity; and Ideal: ≥150 min/wk of moderate physical activity; or ≥75 min/wk of vigorous physical activity; or ≥150 min/wk of combined moderate and vigorous physical activity.

^d^ Diet quality was assessed by evaluating ideal consumption levels for 5 dietary components (fruits and vegetables:$\geq$4.5 cups/day; fish:> 3.5 ounces, twice per week; sodium: <1500mg/day; sugary beverages: <450 kcal/wk and whole grains: $\geq$ 3 servings/day). The food frequency questionnaire was then categorized into three groups based on the number of components that meet the guideline. Poor: 0-1 components; Intermediate: 2-3 components; Ideal: 4-5 components.

^e^ Poor: > 7 drinks/wk for women and >14 for men; Intermediate: zero drinks/wk; and Ideal: 1-7 drinks/wk for women and 1-14 for men.

# **Supplemental Table S2.** Effect Modification by Sex, Age, Education, and Income in the Association Between Continuous Social Integration Score and Mortality, Jackson Heart Study, 2000-2018

|  | HR | 95% CI | | p-value |
| --- | --- | --- | --- | --- |
| **Sex Interaction Model** |  |  |  |  |
| Social integration | 0.95 | 0.91 | 0.99 | 0.007 |
| Male (vs. Female) | 2.26 | 1.43 | 3.59 | 0.000 |
| Social integration*Sex | 0.97 | 0.92 | 1.02 | 0.246 |
| **Age Interaction Model** |  |  |  |  |
| Social integration | 0.96 | 0.93 | 0.99 | 0.008 |
| Age ≥ 55 yr (vs. Age < 55 yr) | 0.25 | 0.15 | 0.42 | 0.000 |
| Social integration*Age | 0.96 | 0.90 | 1.03 | 0.276 |
| **Education Interaction Model** |  |  |  |  |
| Social integration | 0.91 | 0.88 | 0.95 | 0.000 |
| Education yr ≤ 13 (vs. Education year >13) ^a^ | 0.74 | 0.47 | 1.17 | 0.202 |
| Social integration*Education | 1.04 | 0.98 | 1.10 | 0.184 |
| **Income Interaction Model** |  |  |  |  |
| Social integration | 0.90 | 0.86 | 0.94 | 0.000 |
| Lower income (vs. Higher) | 1.11 | 0.67 | 1.86 | 0.681 |
| Social integration*Income | 1.04 | 0.98 | 1.10 | 0.194 |

Models are adjusted for sex, age, educational status, income, depressive symptom measured by CES-D.

^a^ “Education yr ≤ 13” combined “Less than high school” and “High school graduate/GED” in Table 1. “Education year >13” is “Vocation/trade, or college” in Table 1.

# **Supplemental Table S3.** Hazard Ratios for Associations of Social Integration With All-Cause Mortality After Excluding Participants who Died within 4 years from Baseline. Jackson Heart Study, 2000-2018 (n=5,116, n_death_ = 1,065)

|  | Person-years | Cases | Model 1^a^ | | | Model 2^b^ | | | Model 3^c^ | | | Model 4^d^ | | | Model 5^e^ | | |
| --- | --- | --- | --- | --- | --- | --- | --- | --- | --- | --- | --- | --- | --- | --- | --- | --- | --- |
| Exposure |  |  | HR | 95%CI | | HR | 95%CI | | HR | 95%CI | | HR | 95%CI | | HR | 95%CI | |
| **Berkman-Syme Social Network Index** |  |  |  |  |  |  |  |  |  |  |  |  |  |  |  |  |  |
| Highly Socially Integrated | 19,950 | 214 | 0.71 | 0.61,0.82 | | 0.76 | 0.65, 0.90 | | 0.76 | 0.65, 0.90 | | 0.75 | 0.64, 0.89 | | 0.78 | 0.67, 0.93 | |
| Moderately Socially Integrated | 19,105 | 231 | 0.85 | 0.73,0.99 | | 0.88 | 0.75, 1.03 | | 0.88 | 0.75, 1.03 | | 0.88 | 0.75, 1.03 | | 0.89 | 0.76, 1.04 | |
| Moderately Socially Isolated (Ref.) | 20,354 | 301 | 1.00 | Ref | | 1.00 | Ref | | 1.00 | Ref | | 1.00 | Ref | | 1.00 | Ref | |
| Highly Socially Isolated | 2,720 | 35 | 1.63 | 1.19, 2.24 | | 1.28 | 0.92, 1.78 | | 1.23 | 0.89, 1.70 | | 1.23 | 0.89, 1.70 | | 1.15 | 0.83, 1.59 | |
|  |  |  | p ^f^ <0.001 | | | p ^f^ <0.001 | | | p ^f^ <0.001 | | | p ^f^ <0.001 | | | p ^f^ =0.001 | | |

Model 1^a^ Age adjusted

Model 2^b^ Model 1 + sociodemographic conditions (sex, income, and educational status)

Model 3^c^ Model 2 + depressive symptom measured by CES-D

Model 4^d^ Model 3 + health conditions (hypertension, diabetes, cholesterol, cardiovascular disease, and cancer)

Model 5^e^ Model 4 + health behaviors (physical activity, diet, smoking behaviors, alcohol consumption and body mass index)

p ^f^: P for trend

Notes. CI=Confidence Interval; Ref.=Reference; HR=Hazard Ratio; CES-D=Center for Epidemiologic Studies Depression Scale

# **Supplemental Table S4**. Hazard Ratios for Associations of Social Integration With All-Cause Mortality After Excluding Participants who had Cardiovascular Disease or Cancer at Baseline. Jackson Heart Study, 2000-2018 (n=4,470, n_death_ = 837)

|  | Person-years | Cases | Model 1^a^ | | | Model 2^b^ | | | Model 3^c^ | | | Model 4^d^ | | | Model 5^e^ | | |
| --- | --- | --- | --- | --- | --- | --- | --- | --- | --- | --- | --- | --- | --- | --- | --- | --- | --- |
| Exposure |  |  | HR | 95%CI | | HR | 95%CI | | HR | 95%CI | | HR | 95%CI | | HR | 95%CI | |
| **Berkman-Syme Social Network Index** |  |  |  |  |  |  |  |  |  |  |  |  |  |  |  |  |  |
| Highly Integrated | 17,616 | 167 | 0.71 | 0.60, 0.85 | | 0.78 | 0.65, 0.94 | | 0.78 | 0.65, 0.94 | | 0.78 | 0.65, 0.93 | | 0.82 | 0.68, 0.99 | |
| Moderately Integrated | 16,776 | 178 | 0.89 | 0.74, 1.06 | | 0.93 | 0.77, 1.11 | | 0.93 | 0.77, 1.11 | | 0.93 | 0.77, 1.11 | | 0.93 | 0.77, 1.12 | |
| Moderately Isolated (Ref.) | 17,693 | 244 | 1.00 | Ref | | 1.00 | Ref | | 1.00 | Ref | | 1.00 | Ref | | 1.00 | Ref | |
| Highly Isolated | 2,340 | 29 | 1.83 | 1.26, 2.68 | | 1.48 | 1.00, 2.18 | | 1.46 | 0.99, 2.16 | | 1.50 | 1.03, 2.20 | | 1.39 | 0.94, 2.05 | |
|  |  |  | p ^f^ <0.001 | | | p ^f^ <0.001 | | | p ^f^ <0.001 | | | p ^f^ <0.001 | | | p ^f^ =0.006 | | |

Model 1^a^ Age adjusted

Model 2^b^ Model 1 + sociodemographic conditions (sex, income, and educational status)

Model 3^c^ Model 2 + depressive symptom measured by CES-D

Model 4^d^ Model 3 + health conditions (hypertension, diabetes, cholesterol, cardiovascular disease, and cancer)

Model 5^e^ Model 4 + health behaviors (physical activity, diet, smoking behaviors, alcohol consumption and Body Mass Index)

p ^f^: P for trend

Notes. CI=Confidence Interval; Ref.=Reference; HR=Hazard Ratio; CES-D=Center for Epidemiologic Studies Depression Scale

# **Supplemental Table S5**. Hazard Ratios for Associations of Social Integration With All-Cause Mortality Without Imputing Missing in the Social Integration Composite, Jackson Heart Study, 2001-2018 (n=4,224, n_death_ = 911)

|  | Person-years | Cases | Model 1^a^ | | | Model 2^b^ | | | Model 3^c^ | | | Model 4^d^ | | | Model 5^e^ | | |
| --- | --- | --- | --- | --- | --- | --- | --- | --- | --- | --- | --- | --- | --- | --- | --- | --- | --- |
| Exposure |  |  | HR | 95%CI | | HR | 95%CI | | HR | 95%CI | | HR | 95%CI | | HR | 95%CI | |
| **Berkman-Syme Social Network Index** |  |  |  |  |  |  |  |  |  |  |  |  |  |  |  |  |  |
| Highly Integrated | 20,030 | 246 | 0.71 | 0.61, 0.82 | | 0.76 | 0.65, 0.90 | | 0.76 | 0.65, 0.90 | | 0.75 | 0.64, 0.89 | | 0.75 | 0.67, 0.93 | |
| Moderately Integrated | 19,194 | 268 | 0.85 | 0.73, 0.99 | | 0.88 | 0.75, 1.03 | | 0.88 | 0.75, 1.03 | | 0.88 | 0.75, 1.03 | | 0.89 | 0.76, 1.04 | |
| Moderately Isolated (Ref.) | 20,468 | 351 | 1.00 | Ref | | 1.00 | Ref | | 1.00 | Ref | | 1.00 | Ref | | 1.00 | Ref | |
| Highly Isolated | 2,751 | 46 | 1.63 | 1.19, 2.24 | | 1.28 | 0.92, 1.78 | | 1.26 | 0.90, 1.76 | | 1.23 | 0.89, 1.70 | | 1.15 | 0.83, 1.59 | |
|  |  |  | p ^f^ <0.001 | | | p ^f^ <0.001 | | | p ^f^ <0.001 | | | p ^f^ <0.001 | | | p ^f^ =0.001 | | |

Model 1^a^ Age adjusted

Model 2^b^ Model 1 + sociodemographic conditions (sex, income, and educational status)

Model 3^c^ Model 2 + depression symptom measured by CES-D

Model 4^d^ Model 3 + health conditions (hypertension, diabetes, cholesterol, cardiovascular disease, and cancer)

Model 5^e^ Model 4 + health behaviors (physical activity, diet, smoking behaviors, alcohol consumption and Body Mass Index)

p ^f^: P for trend

Notes. CI=Confidence Interval; Ref.=Reference; HR=Hazard Ratio; CES-D=Center for Epidemiologic Studies Depression Scale

# **Supplemental Table S6**. **Hazard Ratios for Associations of Social Integration With All-Cause Mortality, when “Highly isolated” and “Moderately isolated” are combined, Jackson Heart Study, 2000-2018 (N=5,306)**

|  | Person-years | Cases | Model 1^a^ | | | Model 2^b^ | | | Model 3^c^ | | | Model 4^d^ | | | Model 5^e^ | | |
| --- | --- | --- | --- | --- | --- | --- | --- | --- | --- | --- | --- | --- | --- | --- | --- | --- | --- |
| Exposure |  |  | HR | 95%CI | | HR | 95%CI | | HR | 95%CI | | HR | 95%CI | | HR | 95%CI | |
| **Berkman-Syme Social Network Index** |  |  |  |  |  |  |  |  |  |  |  |  |  |  |  |  |  |
| Highly integrated | 20,031 | 246 | 0.67 | 0.58, 0.77 | | 0.73 | 0.63, 0.85 | | 0.73 | 0.63, 0.85 | | 0.72 | 0.62, 0.84 | | 0.76 | 0.66, 0.89 | |
| Moderately integrated | 19,195 | 268 | 0.83 | 0.72, 0.96 | | 0.88 | 0.76, 1.01 | | 0.88 | 0.76, 1.01 | | 0.88 | 0.76, 1.01 | | 0.89 | 0.77, 1.04 | |
| Highly or Moderately isolated | 23220 | 397 | 1.00 | Ref | | 1.00 | Ref | | 1.00 | Ref | | 1.00 | Ref | | 1.00 | Ref | |
| *P for trend* |  |  | p ^f^ <0.001 | | | p ^f^ <0.001 | | | p ^f^ <0.001 | | | p ^f^ <0.001 | | | p ^f^ =0.001 | | |

CI=Confidence Interval; Ref.=Reference; HR=Hazard Ratio; CES-D=Center for Epidemiologic Studies Depression Scale

Model 1^a^ Age adjusted

Model 2^b^ Model 1 + sociodemographic conditions (sex, income, and educational status)

Model 3^c^ Model 2 + depression symptom measured by CES-D

Model 4^d^ Model 3 + health conditions (cholesterol, diabetes, hypertension, cardiovascular disease, and cancer)

Model 5^e^ Model 4 + health behaviors (smoking, physical activity, diet, alcohol consumption, and body mass index)

# **Supplemental Text 1.** Description of Health Conditions and Health Behavior-related Factors

Glucose, HbA1c, and cholesterol were assayed from the blood draw obtained at exam 1 (13). Specifically, fasting plasma glucose (FPG) was measured from fasting venous blood samples with standard glucose oxidase colorimetric methods, and participants were considered as having diabetes if they had FPG$\geq$126 mg/dL, or HbA1c$\geq$6.5%, or use of diabetes medication within 2 weeks prior to the baseline survey. Total cholesterol was measured from fasting venous blood samples and assayed by the cholesterol oxidase method supplied by Boehringer Mannheim Diagnostics on a Roche COBAS Fara analyzer (Indianapolis, Ind), and total cholesterol was categorized as 1) poor, total cholesterol$\geq$240 mg/dL; 2) intermediate, 200-239 mg/dL, or if treated to goal with lipid‐lowering therapy (<200 mg/dL); 3) ideal, < 200 mg/dL and untreated. Using measures of blood pressure obtained by study staff twice at 5-minute intervals at the baseline exam, hypertension was defined as systolic blood pressure (SBP) $\geq$140 mmHg and diastolic blood pressure (DBP) $\geq$90 mmHg or use of blood pressure-lowering medication. History of cancer and cardiovascular diseases, including coronary heart disease, carotid angioplasty (neck arteries), and stroke, was obtained by self-report (yes/no).
 Due to high missingness in Life’s Simple 7 smoking variable that was created by JHS investigators (missing n=904, 19%), we used 2 other smoking variables (i.e., current smoking status, smoked in the past) to create a new 3-category smoking variable: 1) poor, current smoker, 2) intermediate, not a current smoker but smoked in the past, and 3) ideal, never smoked. Physical activity was assessed using a measure of sports and exercise obtained from a validated interviewer-administered questionnaire (14), and was categorized as 1) poor, 0 minutes of moderate or vigorous physical activity, 2) intermediate, 1–149 min/wk of moderate physical activity, 1–74 min/wk of vigorous physical activity, or 1–149 min/ wk of combined moderate and vigorous physical activity, or 3) ideal, ≥150 min/wk of moderate physical activity; or ≥75 min/wk of vigorous physical activity; or ≥150 min/wk of combined moderate and vigorous physical activity. Dietary intake was assessed using a validated 158-item food frequency questionnaire (15), which evaluated ideal consumption levels for 5 dietary components (fruits and vegetables:$\geq$4.5 cups/day; fish:> 3.5 ounces, twice per week; sodium: <1500mg/day; sugary beverages: <450 kcal/wk and whole grains: $\geq$3 servings/day). Diet quality was then categorized according to the number of components for which participants were meeting recommended levels by American Heart Association 1) poor, 0-1 component, 2) intermediate, 2-3 components, 3) ideal, 4-5 components. We also controlled for alcohol consumption, which is not part of Life's Simple 7 but frequently examined as a covariate in the social integration literature. To be consistent with other covariates, we created a 3-category classification using JHS’ alcohol consumption variable assessing the number of drinks consumed weekly: 1) poor, > 7 drinks/wk for women and >14 for men, 2) intermediate, zero drinks/wk, and 3) ideal, 1-7 drinks/wk for women and 1-14 for men. Body mass index (BMI) was computed by dividing weight (kg) by height squared (m^2^), and used as a continuous variable. Height and weight were measured by trained study personnel at the baseline clinic visit. Standing height was measured without shoes and recorded to the nearest centimeter. Weight in light clothing without shoes was measured on a scale.

# **Supplemental Figure S1.** Distribution of the Continuous Social Integration Score, Jackson Heart Study, 2000-2004
